# Theism a New Disease

**Published:** 1888-01

**Authors:** 


### THEISM, A NEW DISEASE.

Attention has recently been drawn to a new nervous disorder said to be especially prevalent in England and America; it is called "Theism," or tea-drinkers' disease. It is said to exist in three stages—the acute, subacute, and chronic. At first the symptoms are congestion of the cephalic vessels, cerebral excitement, and animation of the face. These physiological effects, being constantly provoked, give rise, after awhile, to reaction marked by mental and bodily depression. The tea-drinker becomes impressionable and nervous, pale, subject to cardiac troubles, and seeks relief from these symptoms in a further indulgence in the favorite beverage, which for a time restores to a sense of well-being. These symptoms characterize the first two stages. In chronic cases theism is characterized by a grave alteration of the functions of the heart, and of the vaso-motors, and by a disturbance of nutrition. The patient becomes subject to hallucinations, "nightmares," and nervous trembling. With those who take plenty of exercise, an habitual consumption often may be indulged in with impunity, but with women and young people who follow sedentary occupations this is not the case. The best treatment for theism is said to be indulgence in free exercise, such as walking and open-air life.—*Journal of Am. Med. Association.*

